# Fresh-State Characteristics of Geopolymer Mortars for 3D Printing: Mix Design, Rheology and Early-Age Performance

**DOI:** 10.3390/polym18121479

**Published:** 2026-06-12

**Authors:** İbrahim Türkmen, Enes Ekinci, Fatih Kantarci, Ergun Ekinci, Abdulrahman Ahmad Alyamani, Mehmet Burhan Karakoc, Ramazan Demirboğa, Yasar Ayaz

**Affiliations:** 1 Department of Architectural Engineering, College of Engineering and Advanced Computing, Alfaisal University, Riyadh 11533, Saudi Arabia; 2 Department of Civil Engineering, Faculty of Engineering, Inonu University, 44280 Malatya, Türkiyeyasar.ayaz@inonu.edu.tr (Y.A.); 3 Department of Chemistry, Faculty of Arts and Sciences, Inonu University, 44280 Malatya, Türkiye

**Keywords:** 3D concrete printing, geopolymer, mortar, rheology, workability

## Abstract

The successful application of extrusion-based 3D-printed geopolymer mortars largely depends on precursor chemistry, activator composition, mixture proportions, and fresh-state behavior, which is highly sensitive to time-dependent structural build-up. This review examines the relationships among mix design, geopolymerization chemistry, rheological properties, and printability requirements for 3D-printed geopolymer mortars. Particular emphasis is placed on the effects of precursor type, alkaline activator characteristics, liquid-to-solid ratio, additives, and fibers on flowability, yield stress, viscosity, extrudability, buildability, shape retention, and interlayer bonding. The review further discusses how geopolymerization kinetics influence the evolution of fresh-state properties, the printable time window, and the transition from extrusion to structural stability. In addition, early-age performance is evaluated in terms of setting behavior, green strength development, and layer-interface integrity. Current challenges, including the lack of standardized test methods, limited comparability among published studies, and the complex coupling between material design and process parameters, are also highlighted. Finally, the review identifies key research gaps and proposes future directions for developing robust, printable, and sustainable geopolymer mortar systems for additive manufacturing in construction.

## 1. Introduction

The construction industry has a highly complex structure due to the high degree of interdependence of numerous sub-units on each other’s capabilities during the implementation phase of projects [1]. One of the most significant disadvantages of the construction sector is the difficulty these sub-units face in their efforts to create shared value for the end user [2]. Because many successive work items, starting from project design and extending to painting, are performed by fragmented stakeholders, it is often impossible to achieve a satisfactory result [3,4,5]. These high levels of interdependence cause significant coordination problems, disruptions, and substantial structural damage in construction projects. In this regard, the fragmented and labor-dependent production structure in the construction industry has increased interest in digitally controlled 3D concrete printing technologies that reduce mold usage, speed up production, and improve process safety. However, the successful application of this technology largely depends on the fresh-state behavior, extrudability, buildability, and interlayer bonding performance of the material used. The successful production of various structural elements serving many purposes with this extrusion-based technology has gained significant traction in the scientific community regarding this subject [6]. This technology, which accelerates the building construction process and thus offers significant advantages in terms of labor and time, was reported to be able to reduce production waste, labor costs, and production time by approximately 30–60%, 50–80%, and 50–70%, respectively [7,8,9,10].

Despite these process-related advantages, the sustainability of 3D-printed concrete remains constrained when conventional Portland cement-based mixtures are used. Traditional Portland cement, due to its global standardization and the availability of subtypes to provide solutions for various application practices, serves the sector with an annual production capacity of approximately 6 billion tons [11,12]. The demand for Portland cement, which plays a critical role due to its high accessibility and ability to meet performance targets, is increasing every day. In addition to production-related negative aspects, the use of cement in concrete also leads to several structural problems, such as (i) high heat of hydration, (ii) high shrinkage, and (iii) relatively poor durability [13,14,15]. As a result of all these reasons, the scientific community is making a great effort to develop alternative binder systems that can replace traditional cement.

**Figure 1:**
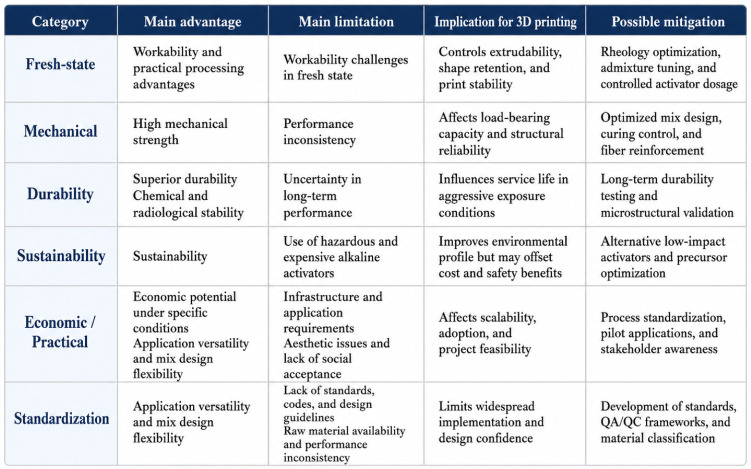
Strengths and limitations of geopolymer binders compared to Portland cement [18].

Introduced by Davidovits in the late 1970s, geopolymers describe a three-dimensional binder material produced through alkali activation of silica and alumina-rich raw materials [16,17]. Although parameters such as raw material type and alkali activator properties affect the characteristics of geopolymer binders, geopolymer binders generally have several advantages and disadvantages compared to their traditional Portland cement counterparts. These are summarized in Figure 1.

**Figure 2:**
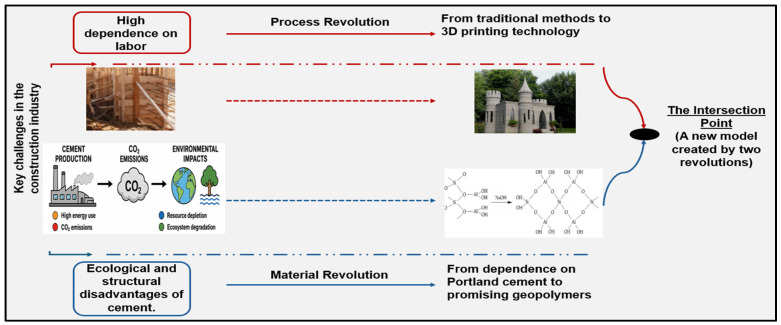
The intersection of process and material revolutions in 3D-printed geopolymer concrete technology.

As seen in Figure 2, extrusion-based 3D-printed geopolymer composites emerge at the intersection of all these developments that fundamentally affect the construction industry. As shown in Figure 2, geopolymer composites produced using extrusion-based 3D printing, a result of the intersection of automation and sustainable material technology, are emerging at the point where all these developments fundamentally impact the construction sector. This approach offers all the benefits of 3D concrete printing technology, along with the high performance and environmentally friendly properties of geopolymers. It should be emphasized that the successful implementation of this integration largely depends on determining the relationship between geopolymer binder chemistry and printability, a thorough understanding of their rheological characteristics, examining the bond structures between 3D-printed concrete layers, and investigating the mechanical and durability properties of the produced samples. Specifically, current research gaps persist in fully understanding the geopolymerization-controlled rheology, the evolution of early-age performance, and the complex interlayer bonding mechanisms required for structural integrity in 3DPGM systems. Accordingly, this review synthesizes current knowledge on 3D-printable geopolymer mortars by focusing on four interconnected dimensions: (i) precursor and activator-based mix design, (ii) geopolymerization-controlled rheology, (iii) printability requirements including pumpability, extrudability, buildability, and interlayer bonding, and (iv) early-age performance. In addition, the review identifies major knowledge gaps and outlines priorities for future research.

## 2. Evolution of 3D-Printed Geopolymer Research: A Bibliometric Perspective

Bibliometric data were obtained from the Web of Science (WOS) Core Collection database, specifically in the “All fields” section, using the keywords “3D printed geopolymer,” “3D concrete printing geopolymer,” “extrusion-based geopolymer,” and “one-part geopolymer 3D printing.” Thus, studies containing these terms in their title, abstract, keywords, or full text were included in the search.

**Figure 3:**
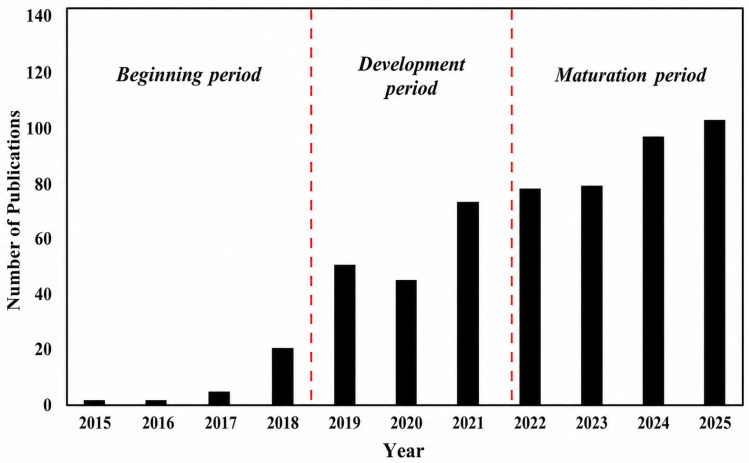
Annual number of publications during the beginning, development, and maturation periods.

The results show that the 3D-printed geopolymer journey, which began in 2015, has become a very popular research topic over the years. As seen in Figure 3, the number of studies observed in 2015 and 2016 (one study each year) reached three-digit numbers by the end of 2025. The findings observed in Figure 3 led us to examine the 3D geopolymer journey in three different periods. The first period, covering 2015–2018, is called the initial period, while the period between 2019 and 2021 is defined as the development period. Finally, the third and final period, covering 2022–2025, is considered the maturation period of the study area.

**Figure 4:**
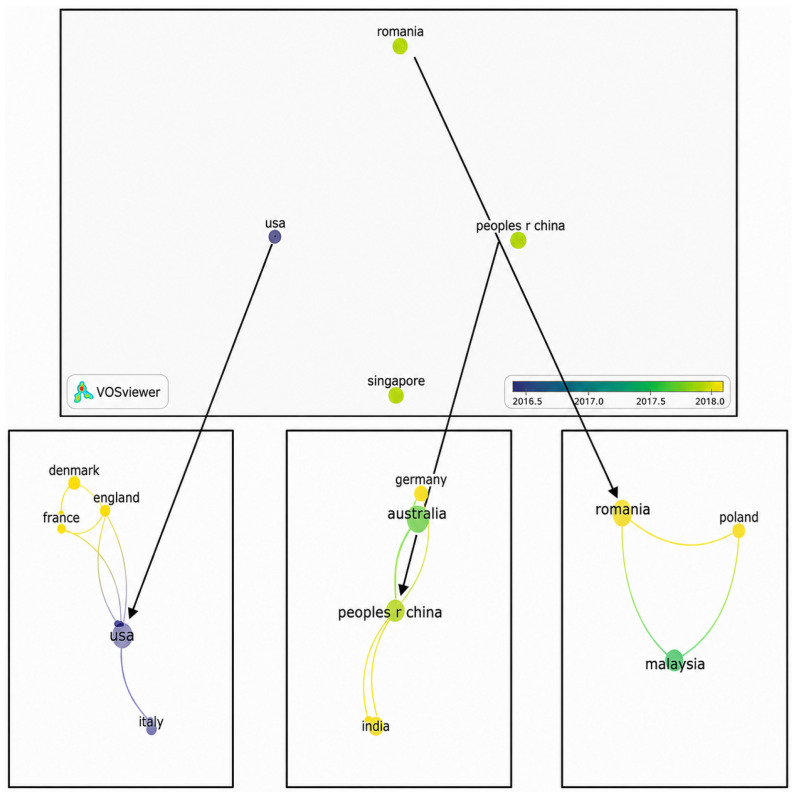
Prominent countries in the initial period covering 2015 to 2018.

**Figure 5:**
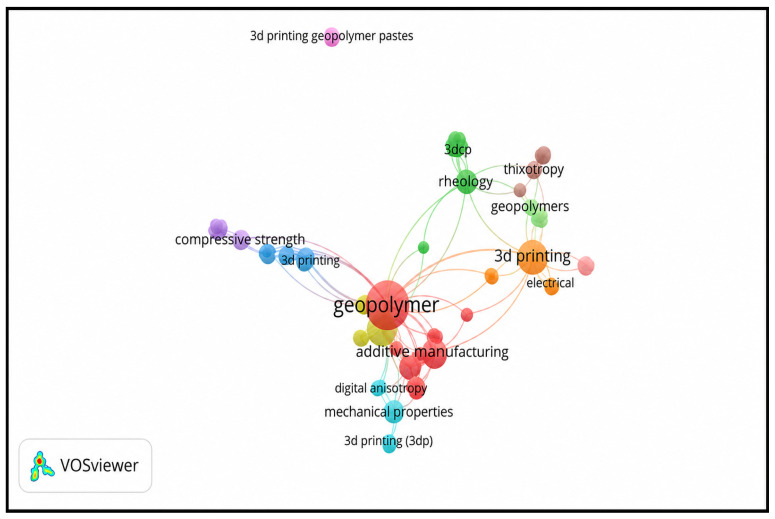
Prominent keywords in the initial period covering 2015 to 2018.

Figure 4 and Figure 5, respectively, show the collaboration network and the keywords where research was concentrated in the field of 3D-printed geopolymer composites during the “initial period” between 2015 and 2018. As seen in Figure 4, a rather sparse and fragmented research network was identified in terms of node sizes and connections. Different node points, led by the People’s Republic of China, USA, and Romania, showed some internal collaborations, but still revealed that strong international consortia could not be established. Another noteworthy finding is that despite Singapore’s strong early publication output, international partnerships were not established, and research was managed by Singaporean research groups. Overall, it should be noted that this fragmented collaboration structure is quite important in interpreting the maturity level of the field. Indeed, these limited connection densities reveal that the studies in the 2015–2018 period were mostly exploratory and feasibility-oriented at the local level, and that an international collaboration agenda has not yet been fully established.

Figure 5 shows that when examining the keyword network for the periods 2015 and 2018, the central concept of geopolymer is strongly associated with “3D concrete printing”, “additive manufacturing”, and “mechanical properties”. Furthermore, the sub-clusters are grouped around “compressive strength” and “rheology, thixotropy, 3D printing”. These findings indicate that in the initial period, studies focused on mechanical properties and rheological parameters that would enable extrusion. Another noteworthy finding is that the concept of “printability” is not central to the network. The absence of “printability” as a central keyword indicated that the field has not yet focused on systematic rheology, open time, buildability, and interlayer bonding issues. This period, which could also be considered the feasibility period, has generally focused on whether 3D-printed geopolymers are possible, particularly in terms of mechanical properties and extrudability. In other words, it was concluded that researchers in the initial period devoted a significant portion of their motivation to exploratory and evidential studies by revealing the process’s operation.

**Figure 6:**
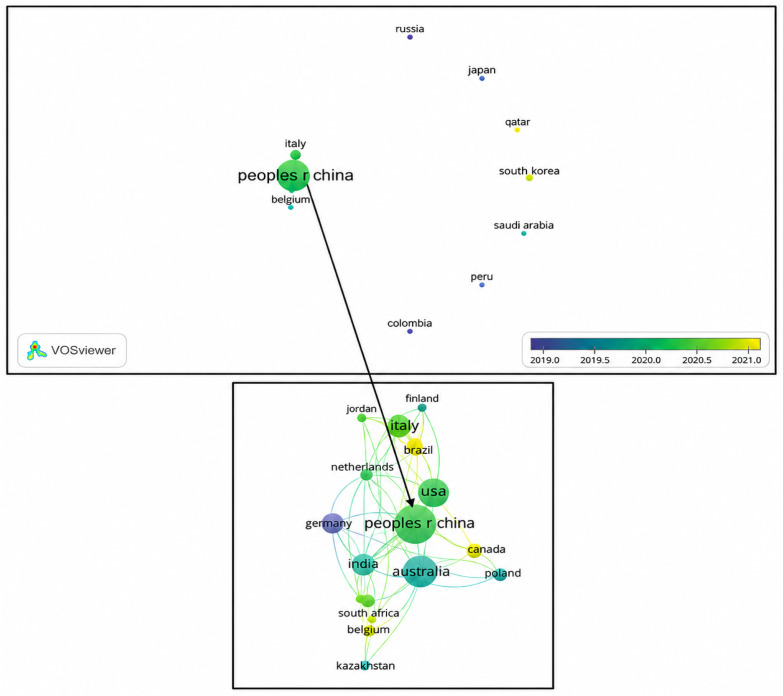
Prominent countries in the development period covering 2019 to 2021.

**Figure 7:**
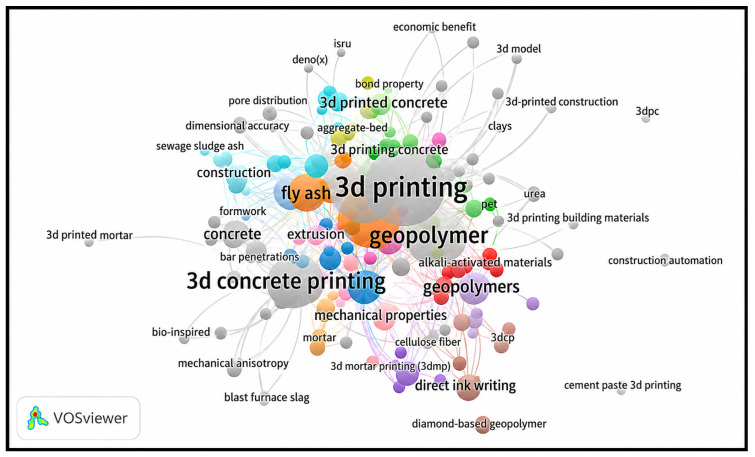
Prominent keywords in the development period covering 2019 to 2021.

Figure 6 and Figure 7, respectively, show the collaboration network and the keywords where research was concentrated in the field of 3D-printed geopolymer composites during the “development period” between 2019 and 2021. Compared to the fragmented and weak collaborations observed in the initial period, more intensive and interconnected research was observed during this period. The most striking feature in Figure 7 is that the People’s Republic of China has become by far the largest node with its numerous international collaboration networks. This situation suggests that China has become an actor not only in terms of broadcast output, but also in shaping the agenda by promoting and directing international collaborations. Overall, it was determined that research between 2019 and 2021 entered a development phase not only in terms of quantity but also in terms of international partnerships and network density. However, it was observed that many countries were still represented by weak node points and remained in an isolated position. This situation showed that while 3D-printed geopolymer research was concentrated in some central country clusters during this period, it remained a local and limited-scale activity for many other countries.

During this period, concepts such as “fly ash,” “extrusion,” “reinforcement,” “cellulose fiber,” “mechanical anisotropy,” “dimensional accuracy,” “economic benefit,” “construction automation,” “thermal insulation,” “alkali-activated materials,” and “bio-inspired” became visible within the network. This situation indicated that research in this period focused more on areas such as the use of different precursor materials, fiber reinforcement, mechanical behavior dependent on the printing direction, dimensional accuracy, economic feasibility, and automation potential. In this context, it was observed that the research focus during this period has shifted from the question of “Can geopolymer mixtures be 3D printed?” to “Which material compositions, reinforcement strategies, and printing parameters can produce more stable, economical, dimensionally accurate, and mechanically reliable structures in 3D-printed geopolymers?” The data obtained showed that the period 2019–2021 can be considered the functionality period for 3D-printed geopolymers. On the other hand, the observation of keywords such as “economic benefit” and “construction automation” indicated that 3D-printed geopolymer research is preparing for a transition from laboratory scale to field application.

**Figure 8:**
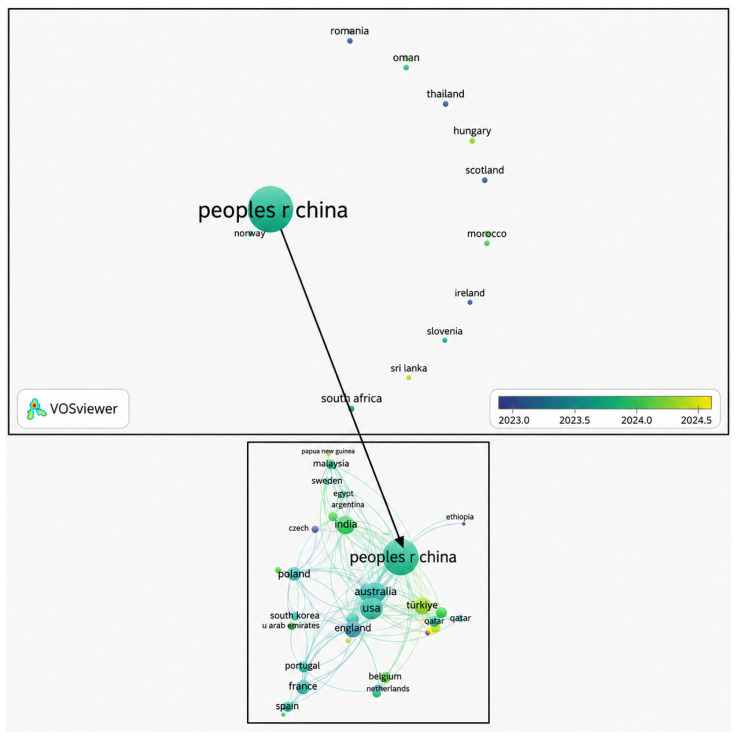
Prominent countries in the maturation period covering 2022 to 2025.

**Figure 9:**
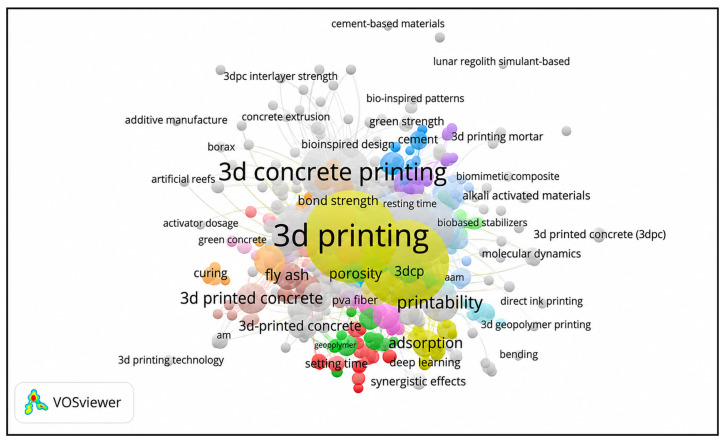
Prominent keywords in the maturation period covering 2022 to 2025.

Figure 8 and Figure 9, respectively, show the collaboration network and the keywords where research was concentrated in the field of 3D-printed geopolymer composites during the “maturation period” between 2022 and 2025. As can be seen in Figure 9, in this third and final period, China has once again maintained its position as the largest node by a wide margin, significantly increasing the number and diversity of its connections compared to previous periods. China’s continued prominence as the largest and most interconnected node demonstrates that knowledge production has not yet achieved a balanced global distribution, and that, despite increasing country diversity, the research landscape remains shaped by core countries. This situation is thought to stem from China’s research infrastructure, R&D investments, large-scale construction sector, and interest in digital construction technologies. Furthermore, countries such as India and Turkey have also expanded their node points and established themselves in a central position.

The keyword network for the period 2022–2025, as seen in Figure 9, shows that 3D-printed geopolymer research is beginning to move beyond the feasibility and basic material testing phase and is evolving into a more mature and process-oriented stage. Additionally, the central positioning of the concepts “3D concrete printing”, “geopolymer”, and especially “printability” in the network indicates that research focus has accelerated during this period towards understanding and controlling the fresh-state behaviors governing the printing process. Remarkably, the concept of “printability” is strongly linked to concepts such as “concrete extrusion”, “green strength”, “interlayer strength”, “porosity”, “absorption”, “bending”, and “compressive strength”.

**Figure 10:**
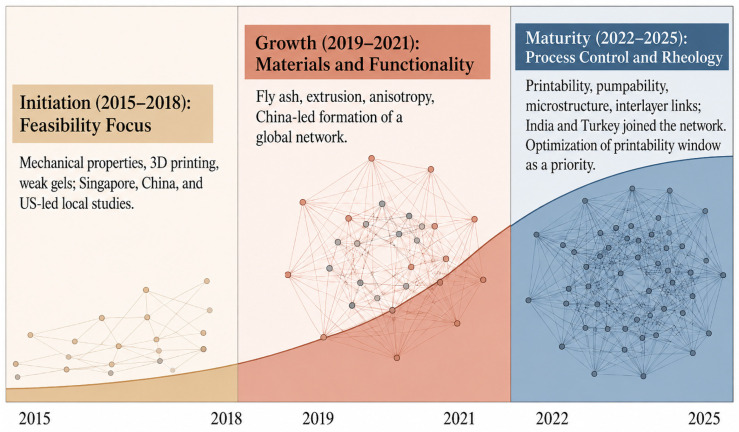
Research evolution: From concept to maturity.

In this paper, the obtained keyword transitions are not considered as an isolated bibliometric observation, but as a conceptual basis for defining the analytical structure of the paper. As illustrated in Figure 10, the strong association of keywords such as “printability” “pumpability” and “extrudability” reveals attempts are revolving from demonstrating the feasibility of 3D-printed geopolymers to understanding the time-dependent fresh-state mechanisms governing printing performance. Therefore, based on this bibliometric evidence, which forms a conceptual bridge to the main body of this article, time-dependent physicochemical transformations during the geopolymerization reaction underlying pumpability, extrudability, buildability, and interlayer bond structures are comprehensively addressed. Subsequently, the review critically examines how these fresh-state properties are affected by precursor reactivity, activator chemistry, liquid-to-solid ratio, additives, temperature, and printing-related process parameters. In summary, it was determined that thematic clustering directly supports the chemistry–rheology–printability framework that structures the present review.

## 3. Geopolymerization-Controlled Rheology and Printability in 3D-Printed Geopolymer Mortars

### 3.1. Geopolymerization and Rheological Evolution

**Figure 11:**
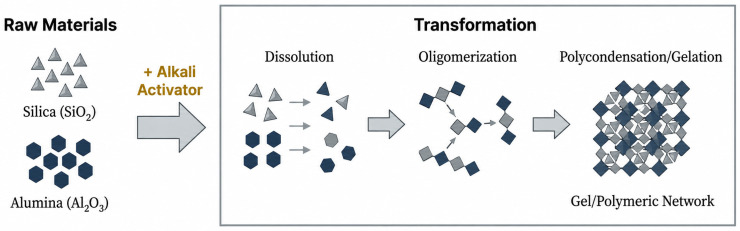
The geopolymerization scheme.

This section discusses the role of geopolymerization in shaping the rheological properties, printability, and interlayer bonding in extrusion-based 3D geopolymer printing. Geopolymerization is an inorganic polymerization process in which natural or waste aluminosilicate raw materials containing silica (SiO_2_) and alumina (Al_2_O_3_) are activated with alkali activators to form high-molecular-weight network structures (gel/polymeric network), as illustrated in Figure 11.

Various physicochemical transformations occurring throughout the process, such as dissolution, oligomerisation, and polycondensation/gelation, cause geopolymerization to exhibit complex rheological behavior in terms of yield stress, viscosity, thixotropy, and interlayer bonding. The ‘printability window’ of the 3D Printed Geopolymer (3DPG) process, which consists of the pumpability, extrudability, and buildability stages, is controlled not only by physical flow characteristics but also, primarily, by rheological changes caused by time-dependent physicochemical transformations; in other words, by the rheochemical mechanism shaped by reaction kinetics. The mixture’s pumpability, its ability to flow through the nozzle without clogging, and its ability to retain its shape when the next layer is applied on top of it are therefore directly related to the evolution of geopolymerization during the process. These fresh-state requirements are basically affected by two simultaneous rheological regimes: dynamic flow within the nozzle and static stability upon deposition.

### 3.2. Dynamic and Static Yield Stress

The printability requirements defined in Section 3.1 are governed by two distinct rheological regimes operating simultaneously. More clearly, these are: a flow regime with low dynamic yield stress occurring under high shear inside the pump/nozzle, and a carrier regime with high static yield stress occurring under a sudden shear drop at the nozzle outlet. On the other hand, while high viscosity makes pumpability and interlayer bonding difficult, low viscosity can cause the mixture to spread and lose its shape. Therefore, for a high-performance extrusion process, it is essential that the system operates within the optimum viscosity range that can maintain the balance between fluidity and shape retention [19,20,21]. This dual regime, which is expected to occur simultaneously, represents the fundamental rheological paradox of 3D printability and requires a perfect optimization study. A critical gap in the literature is the confusion between physical thixotropic recovery and irreversible chemical stiffening. While rapid structural build-up is necessary, if this process is driven primarily by geopolymerization rather than reversible thixotropy, the resulting brittle filament surface can compromise interlayer molecular diffusion. Meanwhile, it should be emphasized that this perfect optimization is quite sensitive to ambient temperature and humidity and is difficult to achieve in large-scale applications. Therefore, rather than finding a static optimum value, it is vital to implement systems that determine an optimum extrusion speed corresponding to the material’s yield stress.

While the three-dimensional networks develop from dimers, trimers, and oligomers to high-molecular-weight polymers as polycondensation progresses, both yield stress and viscosity increase over time, and eventually the flow solidifies. If this situation occurs inside the nozzle, the pump/line may become blocked; if it occurs outside the nozzle, the geometric stability of the structure formed is disturbed, and the layers cannot bond to each other properly. Therefore, while ensuring the flow of the mixture within the nozzle, the structure’s geometry must be preserved at the nozzle outlet through thixotropic recovery, and the transport of upper layers must also be ensured. In summary, it is important to initiate the network development at the right time, i.e., at a point close to the nozzle outlet. Thus, both possible blockages in the nozzle are prevented and strong structural bonds between layers are ensured [22,23].

### 3.3. Printability Window and Interlayer Bonding

A satisfactory 3DPG ‘printability window’ requires a careful balance between the requirements established in Section 3.1 and Section 3.2. This integrity is achieved through interlayer bonding occurring at the interface throughout the printing process, rather than by volumetric strength.

**Figure 12:**
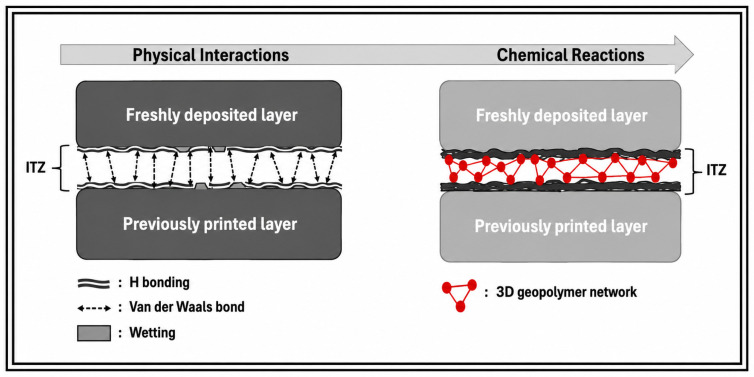
Schematic representation of the interlayer bonding mechanism in 3D-printed geopolymers.

The strength of interlayer bonding depends on variables such as material composition (binder, activator, etc.), fresh-state rheology (viscosity, thixotropy, shape retention), printing parameters (time gap, nozzle geometry, printing speed), and interface microstructure (porosity, surface roughness, contact surface). In interlayer bonding formation, both interlayer physical interactions (Van der Waals, hydrogen bonding, etc.) and chemical reactions (polycondensation) are involved. If the printing time interval elongates, on the one hand, the number of reactive Si–OH/Al–OH groups will decrease due to rapid structural recovery/gradual surface passivation in the lower layer, and on the other hand, the mobility of oligomeric species will decrease due to decreased intra-network bonding/free liquid content. Thus, as a result of insufficient interlayer bonding, fracture will propagate from the interface, toughness will decrease, and the interface porosity will become more permeable, as conceptually presented in Figure 12.

**Figure 13:**
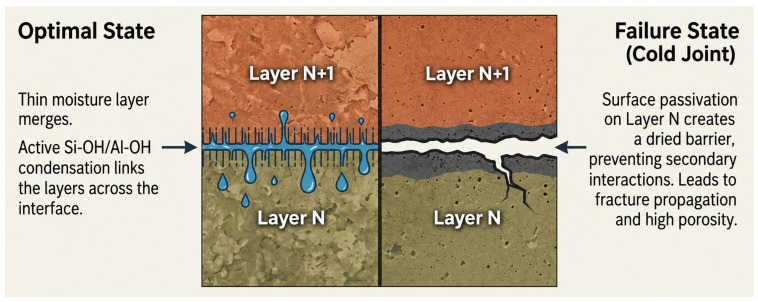
Optimal and failure states in interlayer bonding.

In this case, the material’s geometric stability, mechanical performance, and durability will be limited. The practical indication of this situation is that the structural element transforms into an anisotropic structure, becoming prone to separation along the layer planes rather than behaving as a single piece. To enhance interlayer bonding, strong wetting/physical contact is required beforehand, followed by a period of reactivity (Si–OH/Al–OH condensation) to ensure permanent strength. As schematically illustrated in Figure 13, during the printing process, as the subsequent layer is added before the previous layer has fully hardened, the thin liquid layer/moist surfaces that form between the layers provide a degree of adhesion, thus preparing the ground for the formation of chemical bonds after secondary interactions between the surfaces [24,25].

### 3.4. Effect of Mixture Chemistry on Printability

In general, the pumpability, extrudability, and buildability stages of the extrusion-based 3D printing process are directly related to the chemistry of the geopolymerization mixture and, consequently, to the kinetics it dictates. The type and composition of precursors such as fly ash, metakaolin, and ground granulated blast furnace slag (GGBS), Si/Al ratio, alkali activator type (NaOH, KOH, Na_2_SiO_3_, etc.), activator concentration, and liquid/solid ratio play a characteristic role in print quality by affecting the geopolymerisation process and, consequently, the rheological properties.

**Figure 14:**
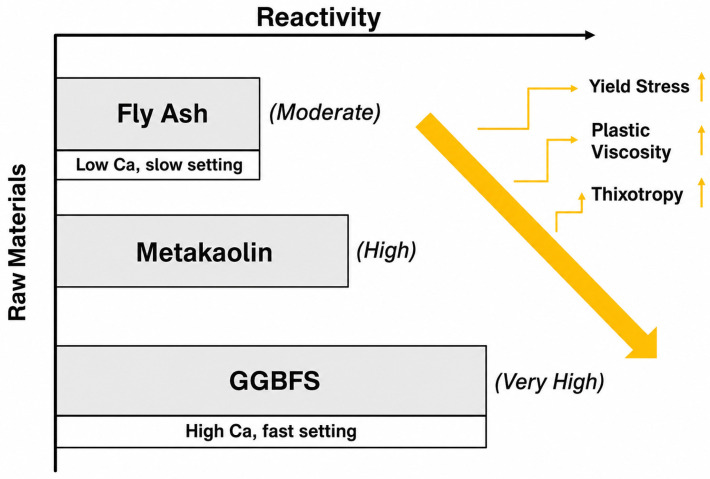
Comparison of rheological properties of different raw materials.

**Figure 15:**
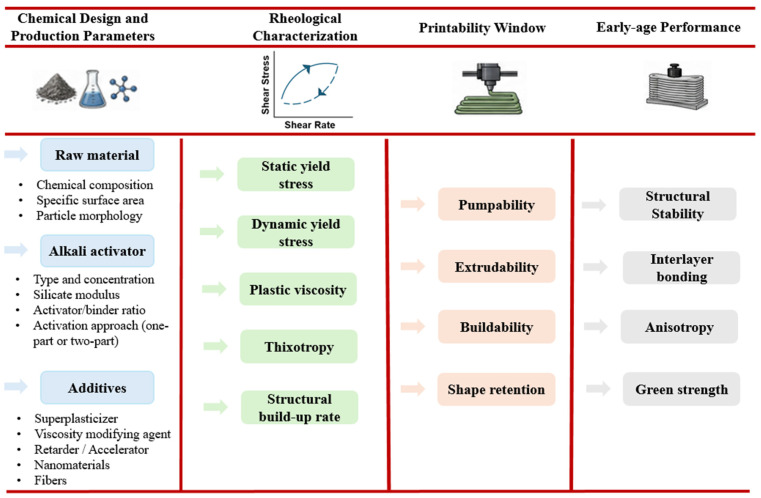
Chemistry, rheology, and printability framework for 3D-printed geopolymer mortars.

Since the precursors that form the main component of the geopolymer have different physical/chemical characteristics, their reactivity, amorphous/crystalline ratios, and dissolution behavior affect the 3D printing performance by shaping the geopolymerization process/rate and thus the rheological behavior. Since polymerization reactions of highly reactive precursors occur faster, the formation of the three-dimensional structure will accelerate and, consequently, the yield stress/viscosity will increase over time. The amorphous structure, which interacts more easily with alkaline activators compared to the crystalline structure, will similarly accelerate three-dimensional structure formation by providing a more reactive environment for geopolymerization. Furthermore, changes in the Si/Al ratio, which cause changes in molecular structure and consequently reactivity, will also affect the print quality by altering the rheology of the system [26,27]. A comparative representation of the rheological behavior of different raw materials is given in Figure 14. Additionally, Figure 15 shows the integrated conceptual framework connecting chemical design, rheological characterization, printability window, and early-age performance in geopolymer mortars produced using 3D printing.

**Figure 16:**
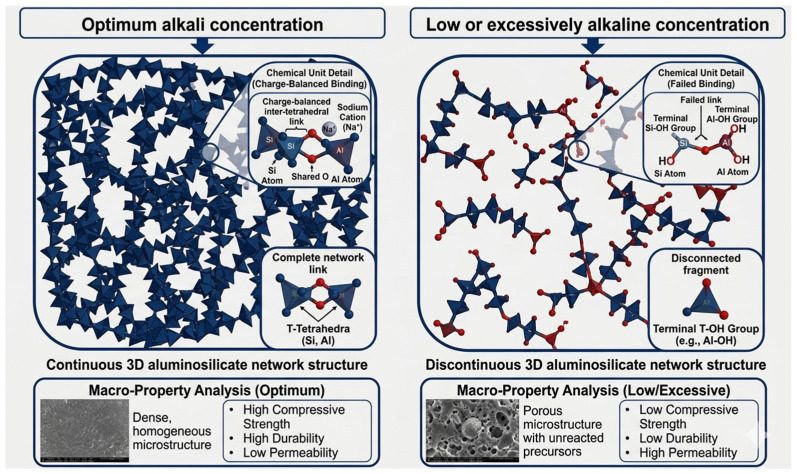
The effect of alkali concentration on the continuity of the 3D aluminosilicate network.

Alkali activators such as NaOH, KOH, and Na_2_SiO_3_, which have different alkalinities and consequently different reactivities, are effective both in dissolving raw material components and in producing reactive monomers that initiate polymerization by regulating the OH^−^ level in the medium. At low alkali activator concentrations, the decrease in the dissolution rate of the raw material, on the one hand, and the reduction in the number of reactive monomers available for polymerization, on the other hand, lead to a decrease in the polymerization rate and consequently to the formation of a weak network structure. Also, silicate activators can alter the rheological behavior of the mixture by providing more reactive silicon to the medium compared with systems using only NaOH. Furthermore, the liquid/solid ratio affects chemical processes and printing characteristics by altering the concentration of the medium. A high L/S ratio causes the mixture to become diluted, reactivity to decrease, and fluidity to increase; thus, buildability decreases, layers spread, and interlayer bonding weakens [7,28]. The effect of alkali concentration on the continuity of the 3D aluminosilicate network is illustrated in Figure 16.

To ensure that 3D-printed geopolymers meet printability requirements, the mix chemistry and production process must be evaluated within specific quantitative limits. Table 1 presents a comparative analysis of the effects of chemical components and production parameters on printability in the 3D geopolymer production process. The data revealed that a universally optimum range could not be achieved for alkali activator chemistry, binder composition, and production process, and that print quality is largely influenced by the chemical composition of the raw material, the activator/binder ratio, and the production parameters.

### 3.5. Temperature, Open Time, and Process Optimization

It is also well known that temperature is an important factor affecting the kinetics of geopolymerization. Increased OH^−^ ion diffusion and solute–solvent interaction with rising temperature accelerate gel formation/network development by increasing solubility, reactivity, and the polycondensation rate. Since temperature not only affects the reaction rate but also indirectly and significantly influences rheological properties, it can be used to regulate these properties during printing. At high temperatures, the open time of the mixture decreases, and extrusion problems occur due to early gelation in the nozzle. Additionally, increasing temperature may cause rapid hardening of the layers and weakening of interlayer bonding by leading to the rapid evaporation of water molecules from the layer surface and by weakening secondary interactions. At low temperatures, the layers may struggle to support their own weight, and thus geometric distortion, spreading, and collapse may be observed in the structure [37].

Beyond chemical kinetics, the ‘printability window’ is limited by the open time; after this time, the material loses its necessary extrusion properties. For instance, variables affecting the geopolymerization rate and the flow performance of the mixture, such as precursor reactivity, activator concentration, and static flow stress, can also alter the open time. Geopolymerization proceeding at an optimal rate prevents cold-joint formation by enabling the desired level of chemical bonding between layers and allows each layer to form a strong bond with the underlying layer. Increasing the polycondensation rate reduces print quality because it causes the mixture to harden rapidly before achieving the desired shape and shortens the setting time. As a result, physicochemical transformations observed during geopolymerization also significantly affect rheochemical behavior, such as open/setting time [20].

**Figure 17:**
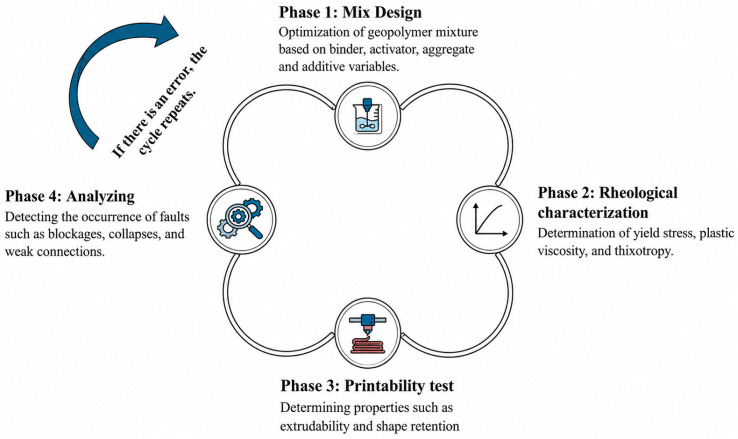
Proposed workflow for optimizing 3D-printed geopolymer.

Consequently, all physical/chemical variables that can affect the geopolymerization process also play a critical role at every stage of the printing process, since they also affect the time-dependent rheological properties of the system. Thus, appropriate formulations should be prepared by carefully evaluating the effect of each factor, and these should be optimized to maximize the efficiency of the 3D printing process. A general workflow for this optimization is presented in Figure 17.

#### Practical Challenges in Hot and Arid Climates and Potential Advantages

Especially in hot and humid climates, the relationship between temperature and open time can become more of a practical limitation than a kinetic equation. Although high temperature and low humidity improve buildability by promoting the geopolymerization reaction, they also accelerate crack and scaling formation by causing evaporation at the layer surface [38]. On the other hand, nozzle clogging can also be observed with the rapid occurrence of the geopolymerization reaction [20]. These results may lead to an increase in shrinkage cracks and a weakening of the bond between layers in the produced samples. Zoude et al. (2024) [39] investigated how the mechanical properties of metakaolin-based geopolymers change with curing moisture, temperature, porosity, and shaping method. In their study, it was noted that samples cured in low-humidity environments exhibit unusually high mechanical properties, and this mechanical performance decreases dramatically when transitioning from a dry to a humid environment. This situation shows that although low relative humidity increases short-term shape stability in 3D-printed geopolymers, it can render reactions insufficient due to rapid water removal and negatively affect long-term performance. Therefore, it should be emphasized that printability parameters determined under laboratory conditions should not be directly transferred to the field scale for 3D-printed geopolymer systems, especially in hot and dry climates. Additionally, future studies should focus on strategies to mitigate the negative effects of adverse parameters such as high temperature, low relative humidity, and wind speed on printability characteristics of geopolymers.

On the other hand, it should not be overlooked that 3D-printed geopolymer applications offer not only limitations but also several potential opportunities. For instance, if the negative effects of rapid surface drying on interlayer bond strength and surface reactivity can be minimized, it may be possible to produce samples with faster early strength and shorter drying times. Furthermore, the use of 3D printing technology can contribute to occupational health and safety by alleviating the suffering of workers exposed to harsh field conditions during hot seasons.

### 3.6. The Effect of Fiber Addition on Fresh State Characteristics and Printability

The use of fibers to improve the mechanical properties of 3D-printed geopolymers has become an increasingly common research topic. However, the effects of the fibers used on 3D-printed geopolymer samples are not limited solely to strength increases. In addition to strength increase, fibers have significant positive and negative effects on the rheological behavior of fresh mixtures. For example, fiber addition increases the viscosity and static yield stress of the mixture, thus providing superior buildability [40]. However, with the increase in viscosity and static yield stress values, the mixture will become more difficult to pass through the nozzle, and extrudability will be negatively affected. On the other hand, using high proportions of fibers can also cause agglomerations, leading to issues such as flow and filament discontinuity. Furthermore, in research focusing on the application of 3D printing, it was determined that fibers tend to align with the direction of the printing flow [41,42]. While this directional alignment can significantly enhance crack-bridging capacity and ductility along the print path, it simultaneously induces mechanical anisotropy, necessitating a precise balance between fiber-induced reinforcement and workability [43]. Therefore, this bidirectional effect of fibers on the 3D printing system is vital for the fresh and hardened properties of 3D-printed geopolymer. Thus, in fiber-reinforced 3D geopolymer blends, the primary goal should be to establish an optimum balance between fiber quantity and fluidity, extrudability, buildability, shape retention, and mechanical properties. As shown in Table 2, data from various studies have determined that fiber addition has both positive and negative effects and is a crucial design parameter.

## 4. Fresh-State Requirements for 3D-Printable Geopolymer Mortars

**Figure 18:**
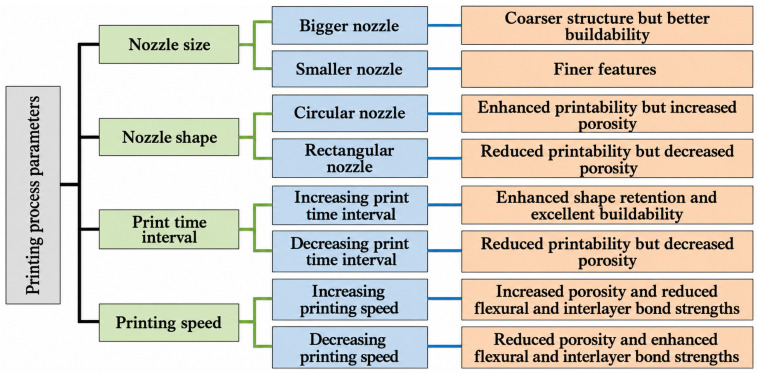
Schematic representation of the influence of printing process parameters on rheological and structural characteristics [20].

The production of geopolymer suitable for 3D printing is a relatively new and promising area in construction. However, the most significant challenge is developing suitable mixtures to achieve concrete that does not slump and can self-compact. In principle, materials suitable for concrete printing should exhibit a balanced combination of static yield stress, viscosity, and thixotropic behavior, as defined in Section 1. Excessively low viscosity is not always beneficial, as it may reduce filament stability, increase segregation risk, and adversely affect layer geometry. However, unlike OPC-based systems, alkali-activated geopolymer binders do not always exhibit such an optimal rheological balance [48]. A schematic representation of the influence of printing process parameters on rheological and structural characteristics and the iterative process for preparing printable concrete mixes is given below (Figure 18).

### 4.1. Comparison of One-Part and Two-Part Geopolymer Systems in Extrusion-Based 3D Printing

Extrusion-based processes necessitate a critical comparison of one-part and two-part geopolymer systems due to the activator effect on manufacturing safety and rheological and mechanical properties [19]. In two-part geopolymers, the use of alkali solutions enables more efficient control of rheological properties and printability, but significantly limits application practices [49]. In contrast, one-part geopolymers, which adopt a “just add water” approach, have become more attractive in field-scale applications due to their user-friendliness, logistical efficiency, and high occupational safety [50]. However, the behavior of one-part geopolymers in the fresh state is determined by parameters that affect the dissolution kinetics of solid activators (activator type and dosage, dissolution rate, water requirement). This leads to unpredictable reaction rates, rapid setting, and limited open time [20,51]. For instance, in a previous study investigating the 3D printability of one-part alkali-activated materials, increasing the activator content from 10% to 20% resulted in a higher yield strength due to a higher pH and ionic strength of surface charges [52]. Furthermore, ensuring complete dissolution of the solid activator before extrusion of one-part geopolymers is crucial for buildability and interlayer bonding structure. For example, it was determined that a fresh mixing time of at least 15 min for FA and GGBS-based one-part geopolymers activated using anhydrous Na_2_SiO_3_ [29]. Therefore, it was emphasized that in one-part geopolymers, simultaneously balancing the high fluidity required for continuous extrusion with structural stability in the layers can create a much more complex optimization problem compared to two-part geopolymers. When these findings are considered together, it was determined that printability in one-part geopolymer systems is not achieved solely by the addition of water, but that the dissolution behavior of solid activators, particle properties, and activator concentration have complex effects on printability. In this context, future efforts should aim to optimize these parameters in an integrated manner and establish a controlled balance between workability, extrudability, and buildability under different environmental conditions.

### 4.2. Experimental Methods Used to Characterize Fresh-State Behavior

When examining the behavior of 3D-printable geopolymer concrete (GPC) in its fresh state, the following experimental methods are generally considered. To quantify these behaviors, the focus is on the development of yield stress and thixotropic recovery, and the optimization of these parameters significantly affects the pumpability, extrudability, and buildability of fresh concrete [53].

In many studies, freshly printed 3D-printable geopolymer mixtures are treated as Bingham or Herschel–Bulkley fluids, and rotational rheometers are used to obtain yield stress and plastic viscosity values [54,55]. Panda and Tan (2018) [56] used a rotational rheometer with a concentric cylinder geometry to characterize fly ash-based geopolymer mortars for 3D printing. The study directly linked extrudability and shape stability to rheological curves and defined the thixotropic open time as the time-dependent period during which the material both maintains its extrudability and retains its shape. Panda et al. (2018) also showed that the change in yield stress and thixotropy over time in GGBS–silica fume-based geopolymer concretes could be related to extrusion quality and buildability [57].

Because rheometers are not always accessible in the field, many studies have used empirical tests such as mini-slump spread, shape retention tests, setting time, and buildability tests to characterize rheology. While the mini-slump or spread diameter test generally aims to remain within a narrow range suitable for extrusion (e.g., ≈130–170 mm), shape retention and buildability tests quantitatively determine height loss, filament deformation, and maximum printable wall height [58,59]. Recent reviews on geopolymer 3D printing summarize these test methods and correlate four key rheological parameters (τs, τd, ηp, and thixotropy) with functional printability metrics such as pumping, extrudability, buildability, and open time [54,55]. On the other hand, one of the practical approaches to evaluating the printable window of geopolymer mortars was encountered in a recent study [60]. This is the flow table test, which involves measuring flow diameters at 30 min intervals (up to 90 min) to determine workability losses over time. With this test, a time-dependent fluidity index is found using the following equation (where *d*_1_ is the maximum spreading diameter, *d*_2_ is the spreading diameter perpendicular to *d*_1_, and *d*_0_ is the 100 mm):

*Γ* = (*d*_1_*d*_2_ − *d*_0_^2^)/*d*_0_^2^ (1)

Similarly, the buildability and shape retention capacities of geopolymers were evaluated using a modified mini-slump test applied at 0, 30, 60, and 90 min. In this method, a load of 600 g was applied to the mixture, and after the load was removed, the amount of slump was measured. The mixture with the lowest average slump value measured along the Z-axis was identified as the mixture with the best buildability and shape retention capacity [61]. Despite some practical benefits, the use of these empirical methods has many limitations and points to a lack of standardization. For example, although the time-dependent variation of the geopolymerization reaction can be measured using these methods, the environment subjected to high shear forces inside the nozzle cannot be successfully simulated. Furthermore, the use of load values not included in the standards for buildability testing and the adoption of arbitrarily chosen time intervals make it difficult to compare the printability of geopolymers with different properties. In this context, it was evaluated that test protocols developed for traditional Portland cement-based systems, which are based on more predictable hydration processes, could not successfully represent the fresh state of geopolymer composites, which react quickly and are more sensitive to temperature changes.

### 4.3. Fresh-State Rheological Requirements Across Pumping, Extrusion, and Post-Deposition Stages

As detailed in Section 3.2, the rheological properties of 3D-printed geopolymers are governed by specific parameters during the pumping and deposition phases. This section examines the necessary numerical ranges for these parameters in detail. In geopolymer concrete applications, pumping performance is highly sensitive to the water/binder ratio, aggregate shape and content, activator viscosity and solid content and morphology, as well as fine additives such as nanoclay and silica fume [57,58]. For example, while an increase in the alkali/binder ratio generally improves workability and fluidity, it was noted that excessive liquid density can lead to segregation and reduced strength [62]. It should be emphasized that an uncontrolled increase in the alkali/binder ratio will not only cause segregation but also lead to excessive ion concentration within the matrix, disrupting the polymerization network and creating a ‘micro-porous’ structure [63]. On the other hand, spherical aggregates increase the fluidity of the mixture by reducing frictional resistance, while angular aggregates have a negative effect on fluidity [15]. However, a necessary balance must be struck between printability and buildability. This is because a high degree of spherical morphology can increase the risk of spreading when layers are stacked on top of each other during the printing process. Another important parameter affecting fluidity is the activator characteristics. Indeed, it has been reported that high NaOH concentrations reduce the workability of the mixture due to its high viscosity, while higher silicate-hydroxide ratios reduce internal friction [64]. However, it should also be considered that the high silicate/hydroxide ratio may have a retarding effect on the setting time, leading to poor adhesion between layers during the printing process. All of this demonstrates that the fluidity of 3D-printable geopolymer mixtures is influenced by a multi-parameter optimization process, rather than being a function of a single parameter.

**Figure 19:**
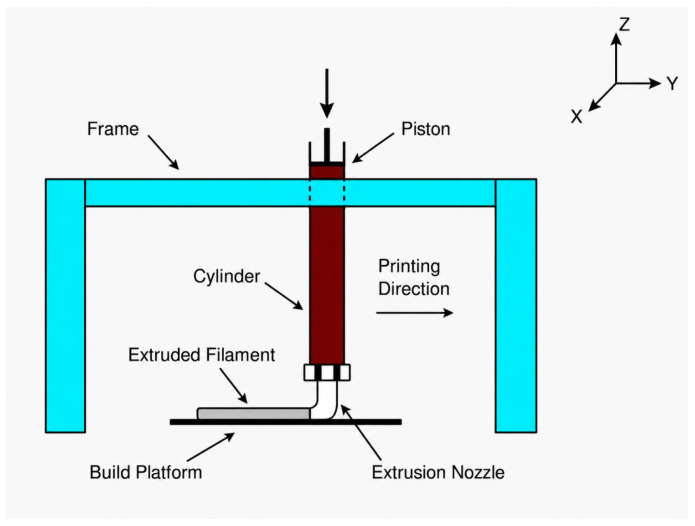
Schematic representation of the extrusion-based 3D-printing process [9].

The systematic method shown in Figure 19 involves extruding the geopolymer paste through a nozzle and then applying it layer by layer to create the designed structure. The mechanical components of such printers are achieved by a kinematic system represented by a gantry or robotic arm [9]. In 3D printing production, the material in the nozzle region can be subjected to high shear rates and extensional flow. The dynamic flow stress must be low enough to allow flow at practical pressures to ensure continuous extrusion, but high enough to form the entire filament [55,56]. For fly ash-based geopolymer concrete production, mixtures with a flow stress of several hundred Pascal (≈0.6–1.0 kPa) and a pronounced thixotropic structure have been found to provide stable extrusion and good dimensional stability, while more fluid mixtures lead to filament spreading (Panda and Tan, 2018). In alkali-activated fly ash- or slag-based binders, microsilica, nanoclay, and viscosity modifiers have been used to maintain mixture homogeneity under pressure while adjusting τd and ηp to achieve sufficient post-deposition structural rigidity [58].

A rapid increase in τs with rest time allows the production of taller wall elements without plastic creep by improving buildability and structural shape retention; however, constructing the structure too quickly may negatively affect the adhesion between different layers [20,56]. In fly ash–GGBS–silica fume-based geopolymer binders, the addition of silica fume significantly increased the viscosity recovery and structural build-up rate of the material, improving shape retention but requiring tighter control of the 3D printing timing [56]. Similar trends have also been reported for alkali-activated slag-based binders, where the addition of nano clay significantly increases thixotropy and τs values, maintaining the material’s pumpability at an acceptable level while improving filament stability and maximum printable structural height (Kondepudi and Subramaniam, 2021) [58]. Buildability models developed for 3D concrete printing emphasize that τs must be high enough to prevent plastic flow and elastic buckling, but low enough to ensure better interlayer adhesion at early ages [53,55]. Geopolymer binders that set very quickly due to high activator concentration or high GGBS content can rapidly reach high levels of τs, but cold joints and low interlayer adhesion can occur in the structure [20,58].

### 4.4. Rheological Ranges and Practical Printability Window

Although experimental methods and structural geometries differ across studies, the literature has identified broadly similar rheological ranges for 3D-printable mixtures. For cement-based 3D concrete printing, printable systems are generally reported to exhibit static yield stress values in the order of 10^2^–10^3^ Pa, whereas the dynamic yield stress is typically lower, often by approximately one order of magnitude, reflecting the thixotropic nature of these materials. In addition, the plastic viscosity is commonly reported to be in the range of several Pa·s at relevant shear rates [53]. These values are often used as indicative reference ranges for geopolymer binders as well, although direct transfer should be made with caution due to differences in precursor characteristics, activator composition, and testing procedures [20,55].

For fly ash-based geopolymer mortars, Panda and Tan (2018) found that mixtures capable of providing both structural shape retention and continuous extrusion have initial yield stress values of several hundred Pascal (∼0.6–1.0 kPa) immediately prior to extrusion, and exhibit pronounced thixotropic behavior after deposition [56]. In studies conducted by the same group on GGBS- and silica fume-based geopolymer binders, they experimentally confirmed that increasing the amount of fine and reactive additives increases both the initial τs and its growth rate; while improving buildability, this narrows the open-time window [57].

For alkali-activated slag-based systems, it was reported that adding nano clay to mixtures designed for extrusion improved thixotropy and static yield stress, thereby increasing filament stability and printable wall height [58]. Other studies on alkali-activated GGBFS-based geopolymer concretes show that mixtures with a mini-slump spread value of approximately 135–165 mm and a static yield stress of approximately 1.8–2.4 kPa can be printed to a height of over 500 mm without slumping, provided they are suitable for pumping [20]. It was also demonstrated that the rheology of high-calcium fly ash geopolymers studied for 3D printing is strongly dependent on activator composition. Changing the NaOH molarity in the range of approximately 8–12 M caused significant changes in slump, rheological parameters, and compressive strength; molarities maintained at medium levels generally provided the most suitable balance between pumpability, extrudability, and buildability [65].

### 4.5. Measurement Protocols and Standardized Reporting of Fresh-State Properties

When referring to printability in 3D-printed geopolymer mortars, the combination of processes such as flow during pumping/extrusion, shape retention in the layer, and subsequent structuring/rapid hardening over time comes to mind. These processes can be evaluated using rheological parameters such as yield stress, apparent/plastic viscosity, thixotropy, elastic modulus (G′), critical shape change, and structuring rate [53,55,66].

RILEM’s technical outputs, which aim to increase reliability in rheological measurements, encourage the reporting of uncertainty sources such as device/geometry effects, wall slip, and data reduction [67]. In the context of digital manufacturing, RILEM TC 276-DFC emphasizes the need to combine fresh-state testing methods and quality control approaches [66,68]. In geopolymer applications, however, rheological parameters obtained due to activator chemistry and early-age reactions can be highly sensitive to measurement time and the applied test protocol [19,20,29]. Therefore, measurement protocols that control shear history and a minimum standardization framework for comparable reporting are required [69,70]. For instance, instead of a single measurement, standardized time-interval measurements at different time intervals (0, 15, 30, 45, and 60 min) should be recommended for reporting rapid time-dependent workability losses in geopolymers. Also, for buildability assessment, qualitative observations should be replaced by standardized load-bearing protocols, such as the application of constant loads to monitor deformation limits over time. Finally, as highlighted in Section Practical Challenges in Hot and Arid Climates and Potential Advantages, geopolymerization kinetics exhibit a much higher sensitivity to temperature compared to conventional cementitious systems. Therefore, the fresh-state performance stability of 3D-printed geopolymers needs to be investigated using temperature-controlled test protocols, and their thermal sensitivity level determined.

### 4.6. Optimization Strategies and Practical Adjustment Framework

In the 3D printing process, the fresh mixture must be simultaneously pumpable, stably extruded from the nozzle, and capable of carrying layers after deposition. These three objectives—pumpability, extrudability, and buildability—are often contradictory: lower flow stress and lower viscosity are desired for pumping/extrusion, while higher static flow stress and faster setting are desired for layer transport [53,66]. The fast reaction kinetics and narrow mixing window in geopolymer mortars further accentuate this conflict [19,20].

In the objective function approach, decision variables such as binder composition (FA/GGBFS/metakaolin), activator type and module, water/binder ratio, sand ratio and granulometry, fibers, and rheology modifiers can be evaluated together with outputs such as pump pressure–flow rate relationship, extrusion continuity, filament spreading/sagging, layer collapse, and rheological parameters changing during printing time. The most frequently reported outputs in the literature for 3D printing production include static/dynamic shear stress (τs, τd), plastic viscosity (ηp), and structuring rate/thixotropy [66,71]. In practice, objectives are often written as “constraints + goals”: (i) no unacceptable maximum pressure or clogging risk in the pump; (ii) no discontinuous flow or filament breakage in the nozzle and stable extrusion at the target speed; (iii) maintaining geometry without collapse up to a certain number of layers; and (iv) sufficient open time and processability during the interlayer period [19,72].

## 5. Conclusions

The reviewed literature demonstrates that the fresh-state performance of extrusion-based 3D-printable geopolymer mortars cannot be interpreted as a simple workability issue. Instead, printability emerges from a time-dependent rheochemical process governed by the coupled evolution of precursor chemistry, activator composition, geopolymerization kinetics, rheological build-up, and printing conditions. Therefore, the central conclusion of this review is that no single mixture parameter can independently define printability. A printable geopolymer mortar must simultaneously satisfy pumpability, extrudability, buildability, shape retention, and interlayer bonding requirements within a narrow and continuously evolving process window.

A key finding is that the chemistry of the geopolymer binder directly determines the fresh-state process window. Precursor reactivity, amorphous phase content, Si/Al ratio, activator type and concentration, silicate modulus, and liquid-to-solid ratio do not merely affect mixture consistency; they control the rate of dissolution, gel formation, network development, and structural build-up. Highly reactive systems can improve early shape stability and green strength, but they may also shorten the open time, increase the risk of nozzle blockage, and reduce the time available for effective interlayer interaction. Conversely, mixtures with lower reactivity may remain pumpable and extrudable for longer periods, but may fail to provide sufficient structural recovery after deposition. This dual behavior shows that the fresh-state design of 3D-printable geopolymer mortars is fundamentally a balance between reaction kinetics and rheological evolution.

The review also reveals that the rheological requirements of geopolymer mortars are strongly stage-dependent. During pumping and extrusion, the material should exhibit sufficiently low dynamic yield stress and viscosity to ensure continuous flow through the nozzle. After deposition, however, the same material must rapidly recover its structure and develop sufficient static yield stress to prevent spreading, deformation, or collapse under subsequent layers. This contradiction represents one of the main challenges in extrusion-based geopolymer printing. Therefore, mixtures optimized only for easy extrusion or only for high buildability are incomplete from a printing perspective. A successful formulation must provide an integrated rheological profile that changes appropriately during the transition from dynamic flow to static stability.

Another important conclusion is that interlayer bonding should not be treated as a secondary hardened-state property, but as a central component of the printability window. The literature indicates that the same mechanisms that improve buildability, such as rapid structural build-up and early stiffening, may negatively affect interlayer bonding if they occur too quickly. In such cases, surface drying, reduced molecular mobility, surface passivation, and loss of wetting ability may limit physical contact and chemical interaction between adjacent layers. Therefore, fresh-state optimization should aim not only to maintain filament geometry, but also to preserve a sufficiently reactive and wettable interfacial state during the layer deposition interval.

Early-age performance should also be interpreted as a continuation of fresh-state evolution rather than as an independent post-printing stage. Setting behavior, green strength development, dimensional stability, and layer-interface integrity are all controlled by the same time-dependent physicochemical transformations that govern the printable window. This means that a mixture that appears printable in a short laboratory test may still fail under real printing conditions if its open time, structural build-up rate, setting progression, or interlayer consolidation is not compatible with the actual printing speed, time gap, nozzle configuration, and environmental conditions.

The review further highlights that temperature and environmental exposure are critical but still insufficiently resolved issues in 3D-printed geopolymer systems. Hot and arid climates may accelerate geopolymerization and improve early buildability, but they may also intensify evaporation, shorten open time, increase shrinkage cracking, promote nozzle clogging, and weaken interlayer bonding. Therefore, printability parameters obtained under controlled laboratory conditions should not be directly transferred to field-scale applications without considering temperature, humidity, wind exposure, and surface moisture loss.

A major gap identified in the literature is the limited comparability of reported fresh-state and rheological data. Published studies differ considerably in terms of precursor type, activator chemistry, liquid content, aggregate morphology, additives, fiber dosage, nozzle geometry, equipment configuration, shear history, measurement time, and testing protocol. As a result, reported values of yield stress, viscosity, thixotropy, open time, buildability, and shape retention should be interpreted as system-specific indicators rather than universal design limits. This lack of comparability is especially critical for geopolymer mortars because their early-age reactions are faster and more sensitive to measurement timing than conventional OPC-based systems.

Accordingly, one of the most important knowledge gaps is the absence of standardized and transferable testing protocols for 3D-printable geopolymer mortars. Future studies should move beyond isolated empirical tests and adopt time-dependent measurement frameworks that combine rheological characterization, flow retention, buildability, shape stability, interlayer bonding, and temperature sensitivity. Standardized reporting of mixture composition, activator chemistry, shear history, test timing, environmental conditions, and printing parameters is essential for meaningful comparison among studies. This is particularly important because current testing approaches developed for OPC-based printable materials may not fully capture the rapid reaction kinetics and rheochemical sensitivity of geopolymer systems.

Overall, the field has moved beyond the initial question of whether geopolymer mortars can be printed. The more important challenge is now how to design geopolymer mixtures whose printable behavior is stable, controllable, scalable, and transferable across different materials, equipment, and environmental conditions. The main conclusion of this review is therefore that robust 3D-printable geopolymer mortars cannot be developed through isolated optimization of individual ingredients. Future progress will depend on establishing integrated composition–rheology–process relationships that simultaneously control flow, structural build-up, open time, interlayer bonding, early-age stability, and field-scale applicability.

## 6. Future Directions for 3D-Printed Geopolymers

This review reveals that the performance of 3D-printed geopolymers is under the complex and combined influence of many parameters. Therefore, for 3D-printed geopolymers to progress from laboratory-scale studies to industrial-scale applications, future research needs to focus on the key research gaps summarized below.

### 6.1. Development of Standardized Rheo-Chemical Kinetic Models

One of the most fundamental gaps in the literature is the lack of standardized predictive models that combine geopolymerization kinetics, which are largely influenced by external environmental conditions, with time-dependent rheological evolution to determine the fresh-state properties of 3D-printed geopolymers. This makes it quite difficult to make meaningful comparisons between different raw material compositions, activator compositions, additives, and printing parameters that affect the rheology of the geopolymer. Furthermore, the existing literature cannot fully simulate the actual flow environment inside the nozzle with rheological tests. Thus, the fresh-state behavior of 3D geopolymers in field conditions in different climatic zones can be predicted with high accuracy, and the production of high-performance 3D-printed geopolymers will be possible.

### 6.2. New Design That Combines Geopolymerization Chemistry and Rheological Evolution

It is necessary to determine the relationship between geopolymerization chemistry and rheological behavior and to create a mix design that directly focuses on this relationship. It is known that the fresh and hardened properties of non-standardized geopolymer binders, such as traditional Portland cement, are largely affected by dissolution, oligomerization, polycondensation, gel formation, and structural recovery. Therefore, by implementing a mix design where the performance of 3D-printed geopolymers is determined by the chemical process, a globally accepted design can be achieved, moving from a trial-and-error mix design to a more universally accepted design. This will enable the implementation of more generalizable and comparable productions for all production parameters.

### 6.3. Strengthening Interlayer Bonding

In 3D-printed geopolymers, interlayer bonding properties are influenced not only by physical contact but also by numerous parameters such as surface moisture, open time, holding time, structural recovery, reactive Si–OH/Al–OH moieties, and fiber alignment. Therefore, future studies should address interlayer bonding as a rheological, chemical, and microstructural interface problem.

### 6.4. Determination of Long-Term Performance

In contrast to current literature data, which generally examines early-age characteristics, future research is recommended to investigate durability problems such as carbonation, chloride penetration, and sulfate attack in detail. This would allow for a more reliable determination of the long-term durability potential of 3D-printed geopolymers under field conditions.

### 6.5. Conducting Sustainability and Life Cycle Analyses

Geopolymers are often considered sustainable or “green” building materials due to their ability to reduce Portland cement use. However, 3D-printed geopolymers require a focus on detailed life-cycle analyses encompassing the broad environmental impacts of different production parameters. This would more concretely demonstrate the feasibility of producing a safe, environmentally friendly, and low-cost building material, thereby increasing industrial interest in this field.

## Figures and Tables

**Table 1:** The effects of material and process parameters on printability in 3D-printed geopolymers.

Parameter	Sample Contents	Key Findings	Reference
Activator Concentration (5, 7.5, 10%), Mixing Time, Use of Retarder (0.5, 1, 1.5%)	GGBFS and FA-based one-part geopolymer activated with anhydrous sodium metasilicate	The addition of 10% solid activator has been shown to improve printing properties. A mixing time of 15 min was determined to be the optimum value for printability. The use of retarder did not lead to significant changes in dynamic rheological properties. Regardless of the activator variables, the static yield stress remained below 5 kPa for 45 min, providing a suitable pumpability window.	[29]
Steel Slag Content (0, 10, 20, 30, 40%)	Steel slag was used in amounts ranging from 0% to 40%.	Yield stress and plastic viscosity increased with increasing steel slag content. Samples containing 10% steel slag exhibit better extrudability, buildability, and mechanical properties compared to other groups. As the steel slag content increased, the dynamic yield stress and plastic viscosity decreased from 1162.11 Pa to 209.70 Pa and 28.24 Pa·s to 11.43 Pa·s, respectively.	[30]
Fiber content (0.1, 0.2, 0.3, 0.4, and 0.5%) and interval time (0, 10, 20, 30, 40, 50 min)	3D printed fiber reinforced geopolymer (3DP-FRG) with PP fibers	Fiber content of 0.5% improved stability and mechanical properties without compromising printability. It was determined that an interval time less than 20 min has no significant effect on the interlayer bonding property. It was found that the fluidity value must be at least 195 mm for trouble-free extrusion. To maintain structural stability, the buildability index must remain above 0.70.	[21]
Waste glass powder usage ratio (0, 10, 20, 30, and 40%)	GGBFS and FA-based geopolymer concrete with waste glass powder	Waste glass powder content below 10% accelerated hydration, increasing buildability. While a waste glass powder content exceeding 10% slowed down hydration, it improved extrudability by extending the processing time. Increasing the waste glass powder ratio from 0% to 40% reduced the static yield strength from 2991.95 Pa to 2748.63 Pa. The dynamic yield stress varied between 162.50 and 167.47 Pa, similar to the reference samples (163.84 Pa).	[31]
GGBFS/FA replacement ratios were changed (25, 50, 75, 100%)	GGBFS, FA, and SF	Replacing GGBFS with FA or SF increased the static yield voltage of AAM systems within 30 min of sedimentation. The addition of FA or SF affected the structural formation rates and intersection times during the flocculation and polycondensation phases. The use of 25% FA and 10% SF for improving 3D printing rheology has yielded the most positive results. Low FA substitution rates increased early-age yield stress and promoted faster structural build-up. Excessive FA content reduced the second-stage thixotropic build-up rate.	[32]
FA/GGBFS ratio (1, 2, and 3), Silicate Modulus (0, 0.3, 0.5, and 1), and sand/binder ratio (0.8, 1, 1.1, 1.2)	Alkali-activated FA/GGBFS	As the sand/binder ratio increased, the interlayer strength improved and drying shrinkage decreased. The static yield stress increased linearly over time. The initial static yield stress, τs,0, ranged from 0.218 to 1.220 kPa, while the structural build-up rate ranged from 0.0675 to 0.342 kPa/min. It was revealed that increasing the sand/binder ratio, decreasing the FA/GGBS ratio, or using a lower Ms activator reduced extrusion capability and accelerated the improvement of buildability.	[33]
SiO_2_/Na_2_O (2.0 and 3.22 and SiO_2_/K_2_O (2.02 and 2.22)	FA and GGBFS-based geopolymer	It was observed that sodium-based activators with a SiO_2_/Na_2_O ratio of 3.22 provide suitable workability and extrusion capability, as well as reasonable strength and high shape retention.	[34]
NaOH concentrations (8, 10, and 12M) and Na_2_SiO_3_/NaOH ratio (1 and 2)	FA and GGBFS-based geopolymer	Increasing the molarity of NaOH improved properties such as shape retention and compressive strength, but negatively impacted setting time and workability. A higher Na_2_SiO_3_/NaOH ratio improved rheological properties. All mixtures were able to produce 250 mm long filaments without blockage, tearing, segregation, or bleeding	[35]
FA (40%), GGBFS, Calcium Carbide slag, and Silica fume (Slag was replaced by calcium carbide slag at (2–16%) or by silica fume at (1–8%), and thickener addition	Different raw material-based geopolymer paste	Reference samples have a yield strength of 24.15 Pa, a consistency coefficient of K = 3.135, and a flow index of n = 1.129. Yield stress increased from 24.15 Pa in the blank paste to 70.26 Pa, 146.36 Pa, and 370.89 Pa with 0.2%, 0.4%, and 0.8% sodium carboxymethyl cellulose, respectively. The use of 0.8% sodium carboxymethyl cellulose increased the extrusion resistance due to its very high yield strength and consistency coefficient.	[36]

**Table 2:** Effects of fiber parameters on performance characteristics of 3D-printed geopolymer.

Fiber Parameters	Key Findings	Reference
Steel fibers of different lengths (6, 8, 10 mm) and different dosages (0.5, 1%)	The flexural strength increased by 282% to 26.1 MPa, while the compressive strength reached 104.5 MPa. Increasing the content of steel fiber or aspect ratio improved the flexural properties.	[44]
Polyvinyl alcohol (PVA) fibers (0, 0.3%, 0.6%, 0.9%, and 1.2%)	Adding 1.2% by volume fiber to the red mud-based geopolymer mixture resulted in more than a threefold increase in extrusion pressure compared to reference samples. PVA fiber had a positive effect on the buildability and shape stability of the 3D printed geopolymer.	[45]
Carbon fiber (0, 0.3, and 0.6%)	The addition of carbon fiber improved shape retention and rheological properties while reducing machinability and extrusion capability.	[35]
Basalt Fiber (0.5 and 1%)	The addition of basalt fibers negatively affected the printing properties, and mortars based on mine tailings/GGBFS produced using basalt fibers could not be extruded. The most suitable mixture content for 3D printing applications was the samples produced with FA/GGBFS and using 0.5% basalt fiber.	[46]
Polyethylene fibers (0.2, 0.4, and 0.6%)	Increasing the fiber volume led to a significant improvement in stacking performance but reduced fluidity.	[47]
Polypropylene fibers (0, 0.1, 0.2, 0.3, 0.4, 0.5%)	The fiber addition negatively affected the printing capacity, interlayer relative humidity, and average interlayer gap width. It was observed that the fiber addition improved stability, mechanical properties, and drying shrinkage, and an optimum fiber content was found as 0.5%.	[21]

## Data Availability

No new data were created or analyzed in this study. Data sharing is not applicable to this article.
